# To the Understanding of Catalysis by D-Amino Acid Transaminases: A Case Study of the Enzyme from *Aminobacterium colombiense*

**DOI:** 10.3390/molecules28052109

**Published:** 2023-02-23

**Authors:** Sofia A. Shilova, Maria G. Khrenova, Ilya O. Matyuta, Alena Y. Nikolaeva, Tatiana V. Rakitina, Natalia L. Klyachko, Mikhail E. Minyaev, Konstantin M. Boyko, Vladimir O. Popov, Ekaterina Yu. Bezsudnova

**Affiliations:** 1 Bach Institute of Biochemistry, Research Centre of Biotechnology of the Russian Academy of Sciences, 119071 Moscow, Russia; 2 Department of Chemistry, Lomonosov Moscow State University, 119991 Moscow, Russia; 3 Complex of NBICS Technologies, National Research Center “Kurchatov Institute”, 123098 Moscow, Russia; 4 Shemyakin-Ovchinnikov Institute of Bioorganic Chemistry of the Russian Academy of Sciences, 117997 Moscow, Russia; 5 N.D. Zelinsky Institute of Organic Chemistry, Russian Academy of Sciences, 119991 Moscow, Russia; 6 Department of Biology, Lomonosov Moscow State University, 119991 Moscow, Russia

**Keywords:** transaminase, D-amino acids, substrate-assisted catalysis, enzymes, structure, X-ray analysis

## Abstract

Pyridoxal-5′-phosphate (PLP)-dependent transaminases are highly efficient biocatalysts for stereoselective amination. D-amino acid transaminases can catalyze stereoselective transamination producing optically pure D-amino acids. The knowledge of substrate binding mode and substrate differentiation mechanism in D-amino acid transaminases comes down to the analysis of the transaminase from *Bacillus subtilis*. However, at least two groups of D-amino acid transaminases differing in the active site organization are known today. Here, we present a detailed study of D-amino acid transaminase from the gram-negative bacterium *Aminobacterium colombiense* with a substrate binding mode different from that for the transaminase from *B. subtilis*. We study the enzyme using kinetic analysis, molecular modeling, and structural analysis of holoenzyme and its complex with D-glutamate. We compare the multipoint binding of D-glutamate with the binding of other substrates, D-aspartate and D-ornithine. QM/MM MD simulation reveals that the substrate can act as a base and its proton can be transferred from the amino group to the α-carboxylate group. This process occurs simultaneously with the nucleophilic attack of the PLP carbon atom by the nitrogen atom of the substrate forming gem-diamine at the transimination step. This explains the absence of the catalytic activity toward (*R*)-amines that lack an α-carboxylate group. The obtained results clarify another substrate binding mode in D-amino acid transaminases and underpinned the substrate activation mechanism.

## 1. Introduction

Chiral amines are important pharmaceutical ingredients and building blocks for drugs. Transamination reaction is an effective biocatalytic route for obtaining optically pure amines, as transaminases demonstrate excellent enantioselectivity and enantiomeric excess of more than 99% [1]. Transaminases (TAs, aminotransferases, EC 2.6.1) are well-established in large-scale chemistry [2,3,4]. These are pyridoxal-5′-phosphate (PLP)-dependent enzymes that catalyze the stereoselective transfer of an amino group from an amino donor (amine or amino acid) to a keto compound (ketone, keto acid, or aldehyde) to form new amino and keto products [5,6,7]. The transamination reaction consists of two half-reactions: the amino group of the first amino substrate is transferred to PLP to produce pyridoxamine 5′-phosphate (PMP) and keto acid (ketone). In the second step, the keto substrate accepts the amino group of PMP to yield a new amino acid (amine) and regenerates PLP [5,8]. The catalytic unit of TAs is a homodimer, with two active sites formed by the amino acid residues of both subunits [9,10,11,12]. Stereospecificity of TAs results from the position of the bound substrate relative to the PLP molecule [5,13,14], which forms Schiff base linkage with the side chain of a lysine residue in the active site, thus dividing the latter into two pockets: the O-pocket (on the phenyl side of PLP) and the P-pocket (on the phosphate side of PLP) [13,15]. The residues lining the O- and P-pockets are involved in substrate binding and determine the substrate specificity of TAs. TAs belong to I and IV fold types according to the organization of PLP-binding fold [16]. TAs of PLP fold type IV are distinguished by both (*L*)-specific and (*R*)-specific amination of keto compounds and include branched-chain L-amino acid TAs (BCATs, EC 2.6.1.46) producing L-amino acids, D-amino acid TAs (DAATs, EC 2.6.1.21) producing D-amino acid and (*R*)-amine: pyruvate TAs producing primary (*R*)-amines (R-TAs, EC 2.6.1.B21) [9,13,17].

**Scheme 1:**
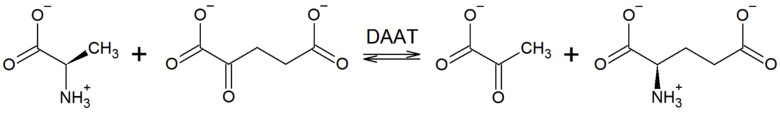
Transamination reaction between D-alanine and α-ketoglutarate, catalyzed by DAAT.

DAATs are found in bacteria and plants [18]. DAATs catalyze synthesis of D-amino acids that in vivo are further involved in antibiotics synthesis, peptidoglycan metabolism, plant growth metabolism, etc. [19,20,21]. In vitro, the benchmark reaction of DAATs is a transamination between D-alanine and α-ketoglutarate (Scheme 1) [22,23].

The properties of several DAATs have been described, namely: DAATs from the bacteria *Bacillus* sp. YM-1 (bsDAAT) [22], *Bacillus sphaericus* (bsphDAAT) [24], *Geobacillus toebii* SK1 (SK1DAAT) [25], *Lactobacillus salivarius* (lsDAAT) [25], *Curtobacterium pusillum* (CpuTA) [26], *Haliscomenbacter hydrossis* (Halhy) [27] and from plant *Arabidopsis thaliana* (atDAAT) [18]. The substrate spectrum of the DAATs includes various D-amino acids and α-keto acids, with CpuTA being also active with primary (*R*)-amines. Crystal structures of some bacterial DAATs are known, these are bsDAAT (PDB ID: 1DAA, 3DAA), CpuTA (PDB ID: 5K3W), and Halhy (PDB ID: 7P7X) and two putative DAATs—from *Burkholderia thailandensis* (btDAAT, PDB ID: 4TM5) and *Mycobacterium tuberculosis* (mtDAAT, PDB ID: 6Q1R).

**Figure 1:**
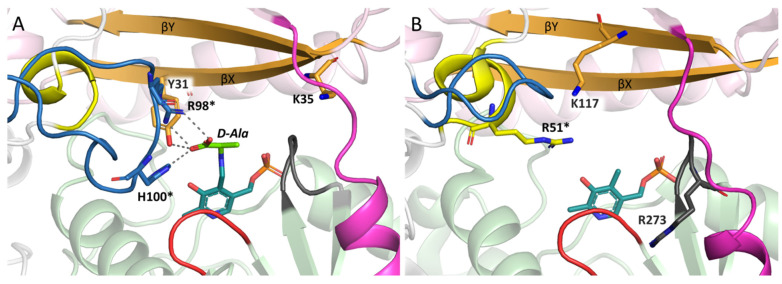
Variants of the active sites in DAATs. (**A**) The active site of bsDAAT in complex with D-alanine (PDB ID 3DAA). (**B**) The active site of CpuTA (PDB ID 5K3W). The small domain of one subunit is colored in light green, the large domain—in light pink, and the adjacent subunit—in grey. The O-pocket α-helix is colored in yellow, the O-pocket loop—in blue, the βX- and βY-strands—in orange, the interdomain loop—in magenta, the β-turn1—in red, and the β-turn2—in black. PLP molecule and important residues are shown in sticks. The O-pocket is to the left from PLP, the P-pocket is to the right from PLP. Residues of the adjacent dimer subunit are marked with asterisks.

Currently, two variants of substrate binding in DAATs are revealed (Figure 1). The first one, canonical (applied for identification of specificity-determining sequence motifs and described by Hőhne et al. [28]), was observed in bsDAAT [11,29,30,31]. The triad of the amino acids in the O-pocket Y31, R98*, and H100* (* here and after indicates residues from the adjacent subunit of the functional dimer) form a carboxylate trap that binds the α-carboxylate group of D-amino acids [31]. Later, Ro et al. proposed that the binding of α-ketoglutarate in bsDAAT is supported by the coordination of the γ-carboxylate group in the P-pocket via K35 [32]. Another non-canonical substrate binding mode has been proposed for CpuTA and Halhy [26,27]. The O-pockets of both TAs lacked a canonical triad, however, possessed two positively charged residues, nonequivalent to the R98* and H100* of bsDAAT—R51*, K117 in CpuTA [26] and R28*, R90 in Halhy [27]. These residues were suggested to bind the α-carboxylate group of substrates D-amino acids [26]. Positively charged residues, nonequivalent to the K35 of bsDAAT, were observed in identical positions in the P-pockets of CpuTA and Halhy—R273 and K241, respectively. However, nothing was suggested about their roles in the binding of the γ-carboxylate group of α-ketoglutarate. 

Herein, we report insights into substrate binding in DAAT from the gram-negative mesophilic bacterium *Aminobacterium colombiense* (AmicoTA) [33]. Using kinetic analysis, we observed the activity of AmicoTA toward different D-amino acids, with D-glutamate being the most specific substrate. Structure-based sequence alignment revealed the identical active site residues between AmicoTA and both canonical and non-canonical DAATs. Thus, both substrate binding modes were allowed in the active site of AmicoTA. To clarify this issue, we investigated AmicoTA using X-ray crystallography and molecular modeling. Finally, molecular dynamic simulations with combined quantum mechanics/molecular mechanics potentials were performed to clarify the molecular mechanism of the formation of gem-diamine from the Michaelis complex that initiates transimination. 

## 2. Results

### 2.1. Structure-Based Sequence Alignment of AmicoTA and Known DAATs 

According to [9,13,28], the active site of DAATs is formed by secondary structural elements (SSE), grouped into the O-pocket and P-pocket (Figure 1). The O-pocket is lined by residues of βX- and βY-strands, β-turn1, and O-pocket α-helix, and is partially capped by the residues of the O-pocket loop. The O-pocket is responsible for the binding of the α-carboxylate group of substrates, and the P-pocket—for the binding of a side group of substrates. Structure-based sequence alignment of homologous DAATs (Figure S1) revealed two groups of DAATs (canonical and non-canonical) differing in the O-pocket organization: DAATs with canonical “carboxylate trap” and DAATs with two R/K residues in the O-pocket (Table 1). The P-pockets in both groups of DAATs do not contain conservative residues. However, in several DAATs P-pockets contain a positively charged R/K residue from βX-strand (bsDAAT, bsphDAAT, SK1DAAT) or β-turn2 (btDAAT, CpuTA, and Halhy). Similar to bsDAAT, these R/K residues could coordinate the γ-carboxylate group of substrate α-ketoglutarate. The interdomain loop in the known structures of DAATs is out of the active site (Figure 1) and seems not to participate in catalysis [27,31,34]. The peculiar feature of canonical DAATs is the high similarity of sequence fragments encoding β-turn1, which borders the entrance to the active site. No similarity is observed among sequence fragments encoding β-turn1s in non-canonical DAATs. β-Turn2, which borders the P-pocket, in both DAAT groups contains Ser and Thr residues in random positions. Finally, the specificity-determining motifs of the two groups of DAATs differ significantly (Table 2). 

Searching for TAs in the genome of bacteria *A. colombiense* revealed a new PLP-dependent DAAT (GenBank ID: WP_013049219)—AmicoTA. The identities of the amino acid sequences of AmicoTA with canonical bsDAAT and non-canonical CpuTA were 28.7% and 31.2%, respectively. The AmicoTA sequence included residues matching to motifs of both DAAT groups (Table 1 and Table 2). While R27 (O-pocket α-helix), F32, T34 (βX-strand), and R88 (βY-strand) residues corresponded to non-canonical DAATs motifs, the K99 and H101 (O-pocket loop) residues matched to canonical DAATs motifs. To shed light on the properties of AmicoTA, the recombinant enzyme was expressed in a soluble form, purified to homogeneity (Figure S2), and characterized in detail.

### 2.2. Substrate Specificity Profile of AmicoTA 

An analysis of half-reactions was performed to find amino donors. Since the overall transamination reaction is the sum of two consecutive half-reactions [8,37], the substrate spectrum (amino donors) of TA can be obtained from the analysis of the TA affinity in half-reactions for compounds with the amino group/keto group (in the absence of the inhibition by the second substrate and the second product) [5,8,14]. The half-reaction analysis of the PLP form of AmicoTA with various amino donors demonstrated the specificity of AmicoTA for various D-amino acids. (Table 3, Figure S3). No activity with L-amino acids and primary amines was observed. The best amino donors were D-glutamate and D-alanine. 

**Figure 2:**
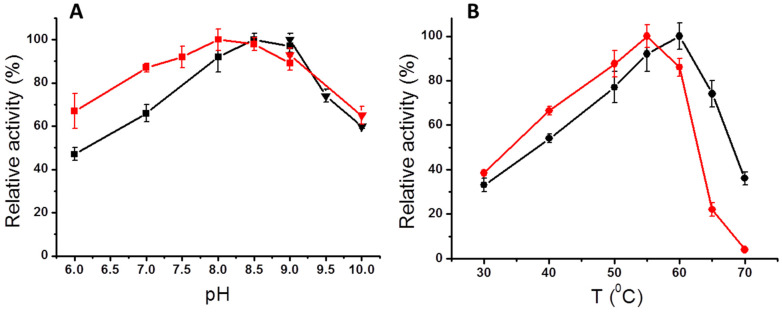
pH- and temperature dependences of AmicoTA activity in the overall transamination reactions between D-alanine and α-ketoglutarate (black) and D-glutamate and pyruvate (red); 100% corresponds to 0.62 ± 0.05 U/mg ((**A**), black), 0.26 ± 0.01 U/mg ((**A**), red), 1.55 ± 0.05 U/mg ((**B**), black) and 0.60 ± 0.03 U/mg ((**B**), red). Black/red squares: mixed buffer 25 mM Tris-HCl and 25 mM K-phosphate, pH 6–9. Black/red triangles: 50 mM CHES buffer, pH 9–10. Bars mean standard deviation.

AmicoTA catalyzed transamination at 30–60 °C and pH 6–10 (Figure 2). The optimal pH and temperature for the reaction between D-alanine and α-ketoglutarate were 8.5–9.0 and 60 °C (Figure 2), respectively. The optimal parameters for the reverse reaction between D-glutamate and pyruvate were pH 8.0 and 50 °C, respectively (Figure 2). Kinetic parameters of the overall transamination reaction between D-alanine and α-ketoglutarate as well as the reverse reaction between D-glutamate and pyruvate are shown in Table 4 (Figure S4). Transamination reaction between D-alanine and α-ketoglutarate in 50 mM CHES buffer, pH 9.0, at 60 °C was assigned as a standard assay.

According to the kinetic analysis of thermostability, the half-life of AmicoTA was about 10 h at 60 °C, 40 h at 50 °C, and 100 h at 40 °C (Figure S5A). In the presence of substrates (operational stability), the half-life of AmicoTA decreased to 10 min at 60 °C, 10 h at 50 °C, and 35 h at 40 °C (Figure S5B). Both experiments were performed at high concentrations of AmicoTA. No enzyme inactivation was observed in the standard assay at lower enzyme concentrations. 

The enantioselectivity of AmicoTA was evaluated in the overall reaction between 4-methyl-2-oxovalerate or phenylpyruvate and D-glutamate as an amino donor. The product yield of D-leucine and D-phenylalanine after 48 h of the reaction at 30 °C achieved 98% and 32%, respectively (Figure S6). The enantiomeric excess of D-leucine and D-phenylalanine was 99.1 and 99.3%, respectively. 

### 2.3. The Overall Structure of AmicoTA

**Figure 3:**
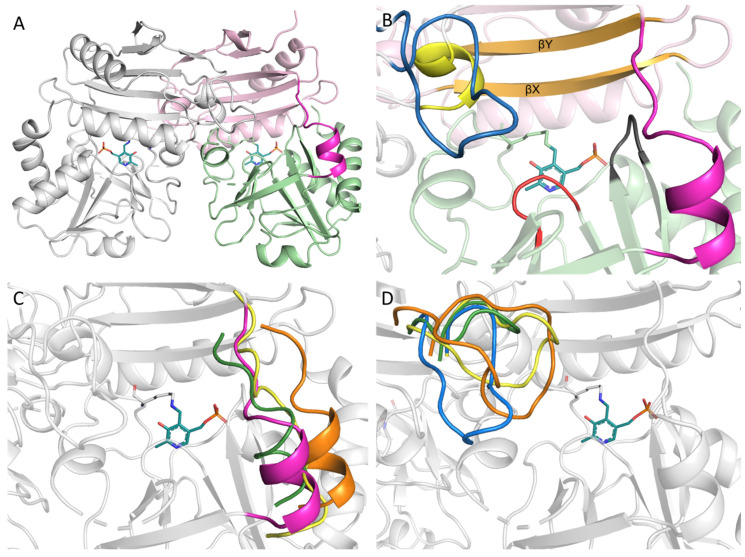
The overall structure of AmicoTA. (**A**) The two subunits in the AmicoTA dimer. In the right subunit, the small domain is shown in light pink, the large domain in light green, the interdomain loop in magenta, and the adjacent subunit in gray. The PLP molecules are shown in sticks. (**B**) The active site of AmicoTA. The important structural elements are shown: βX- and βY-strands (orange), the O-pocket loop from the adjacent subunit (blue), the O-pocket α-helix from the adjacent subunit (yellow), β-turn1 (red), β-turn2 (black). (**C**,**D**) Alignment of the interdomain loops (**C**) and the O-pocket loops (**D**) of AmicoTA (O-pocket loop—blue, interdomain loop—magenta), bsDAAT (orange), CpuTA (green) and Halhy (yellow).

The crystal structure of the holoenzyme was elucidated at 1.9 Å resolution. The asymmetric unit contained two subunits organized in a dimer whose buried area comprised 15% of the total surface area of each subunit. According to gel filtration, AmicoTA is a dimer in solution as well (Figure S7). The organization of the AmicoTA dimer (Figure 3A) corresponded to the functional units of TAs of PLP fold type IV [9,10,11,12,31,38,39], which is in agreement with the general conclusion about the similarity of the overall fold organization among TAs of PLP fold type IV [9,28]. The closest structural homologs of the AmicoTA were BCATs from *Geoglobus acetivorans* and *Archaeoglobus fulgidus* [40], Halhy [27] and R-TA from *A. fumigatus* [12] (Table S1). 

Each subunit of the dimer consisted of two α/β domains: a small domain (residues 1–114) and a large domain (residues 127–275), connected by the interdomain loop (residues 115–126) (Figure 3A). The interdomain loop of AmicoTA (115–126) was out of the active site as in known DAATs (Figure 3B). Two proline residues make the interdomain loop rigid similar to bsDAAT (Table 1). It is fixed on the verge of the active-site cavity by two hydrogen bonds. This arrangement of the interdomain loop seems to be a common feature of DAATs [9,11,34] (Figure 3C). The O-pocket loop (95–109) was also located outside the active site in the AmicoTA dimer (Figure 1B,D), i.e., in an open conformation. Positions of other structural elements in the active site: βX-strand (30–37), βY-strand (85–92), β-turn1 (173–176), and β-turn2 (234–237) were similar to those of known TAs of PLP fold type IV [9,10,11,12]. 

### 2.4. Active Site Organization in the AmicoTA Dimer

**Figure 4:**
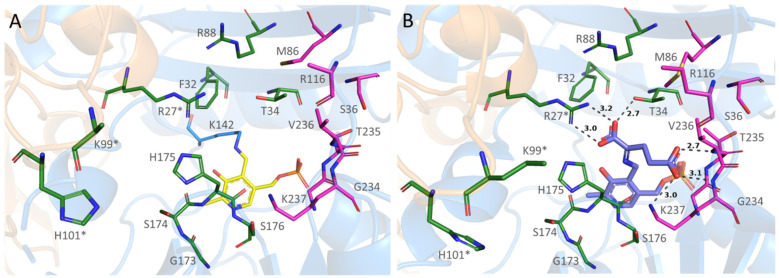
The active site of AmicoTA in (**A**) the holoenzyme with PLP bound to K142, (**B)** in the complex with D-glutamate. Both subunits are shown in cartoon mode: first—in light blue, second—in light orange. Residues of the O-pocket are shown in green, of the P-pocket—in magenta, the PLP molecule is shown in yellow, and the ketimine formed by PLP and D-glutamate is in purple. (*) indicates residues from the adjacent subunit of the functional dimer. Distances are given in Å and depicted with dashed lines.

The AmicoTA dimer contains two identical active sites formed by the residues of two small domains of both subunits and a large domain of one subunit. The PLP molecules are clearly seen in the electron density of both active sites and are bound via Schiff base linkage with K142. The phosphate moiety of PLP has a common to known TAs of PLP fold type IV multiple coordination via hydrogen bonds with the side chains of R51, T198, and T199, backbone nitrogen atoms of T198, T199, and T235 as well as water-mediated hydrogen bonds with T34, T177, R200, and T233. The pyridine ring of PLP is sandwiched by the side chain of L195 and backbone atoms of H175 and S176 (β-turn1) (Figure 4 and Figure S8). Y146 forms a hydrogen bond with the phenyl group of PLP (Figure S8). E172 forms a hydrogen bond with the N1 atom of the pyridine ring of PLP. Noteworthily, E172 forms a direct hydrogen bond with the N1 atom in one subunit (Figure S8A), while it coordinates the N1 atom via the water molecule in the other subunit (Figure S8B). Generally, there is a direct hydrogen bond between the N1 atom of the pyridine ring and the carboxylate group of aspartate or glutamate residue among TAs of PLP fold type IV [10,11,12,26,41].

Considering the conventional division of the active site into O-pocket and P-pocket [9,13,15], the O-pocket of AmicoTA is lined by residues of βX- and βY-strands as well as β-turn1 and O-pocket α-helix. The O-pocket is open: neither the O-pocket loop nor β-turn1 shields it from the solvent. It contains five positively charged residues, which can bind the α-carboxylate group of a substrate: R27*, R88, K99*, H101*, and H175 (Figure 4A). K99* and H101* residues (equivalent to R98* and H100* in canonical bsDAAT) are located on the O-pocket loop and are oriented away from the active site in the holoenzyme. The third residue of the canonical carboxylate trap (Y31 in bsDAAT) is substituted for F32 in AmicoTA; however, the nearby located T34 might bear its function. Although the residues of the canonical triad are mostly in place, the presence of a canonical carboxylate trap is unclear from the holoenzyme structure. Additionally, R27* and R88 from O-pocket α-helix and βX-strand, respectively, together with T34 can organize an alternative carboxylate trap.

The P-pocket of the AmicoTA active site is lined by S36, M86, and ^234^GTVK^237^ residues of the β-turn2 (Figure 4A). K237 forms a positively charged site in the P-pocket. The interdomain loop contains the R116 residue, which might be involved in targeting a negatively charged substrate. Of note, in the holoenzyme structure, the R116 side chain has no electron density. 

### 2.5. Structural Analysis of the Substrates Binding in the Complex of AmicoTA with D-Glutamate

To shed light on the substrate binding mode in AmicoTA, the structure with D-glutamate was obtained at 1.9Å resolution. The RMSD for Cα atoms between subunits of the holoenzyme and the complex with D-glutamate did not exceed 0.4 Å. In the complex D-glutamate is covalently bound with PLP via Schiff linkage (Figure 4B). Considering that the dihedral angle C3-C4-C4′-N4′ is 60° and 40° in subunits A and B, respectively, the adduct between D-glutamate and PLP is a ketimine rather than an aldimine. The hydrogen bond between the phenyl oxygen of the PLP ring and the N4′ atom of Schiff linkage is unobvious. The occupancy of the adduct was found to be 0.7, the rest of the cofactor was in PMP form. Several residues in the active site and PLP changed their orientation upon D-glutamate binding, compared to holoenzyme. The pyridine ring was tilted 15° from its position in the holoenzyme around the N1-C6 bond, and the released side chain of the catalytic lysine moved toward Y146, forming a hydrogen bond between its εNH2 group and OH group of Y146. The α-carboxylate group of D-glutamate formed a salt bridge with the guanidine group of R27* and a hydrogen bond with the hydroxyl group of T34. Backbone nitrogen atoms of V236 and K237 as well as the Nε atom of the latter coordinated the γ-carboxylate group of D-glutamate. 

Both the O-pocket loop and the interdomain loop did not change their conformations upon binding of the substrate. Neither K99* with H101* (analogs of the “carboxylate trap” in bsDAAT) nor R116 was found to participate in the substrate binding. The *si*-face of the ketimine was exposed to the bulk solvent, while the *re*-face oriented to the protein side was shielded from the water molecules. Of note, R88, which is located near R27*, was not involved in the substrate binding; it remained intact, bound by several hydrogen bonds with residues of both subunits of the dimer. The orientation of the side chain of H175 was unfavorable for hydrogen bonding with the α-carboxylate group of D-glutamate, although histidine could be protonated at pH 6.5 (crystallization conditions). Instead, H175 was oriented as in the holoenzyme, which may be a result of crystal soaking in a D-glutamate solution.

To summarize, no canonical “carboxylate trap” was formed when D-glutamate entered the active site. No other “carboxylate trap” was formed by R27*, R88, and T34 as well. The α-carboxylate group of the substrate was coordinated only by R27* and T34. The γ-carboxylate group of D-glutamate was coordinated by the positively charged side chain of K237 and backbone nitrogen atoms of K237 and V236 in the P-pocket. The active site is open to the solvent.

### 2.6. Substrate Binding Modes Revealed by Molecular Dynamic Simulations

**Figure 5:**
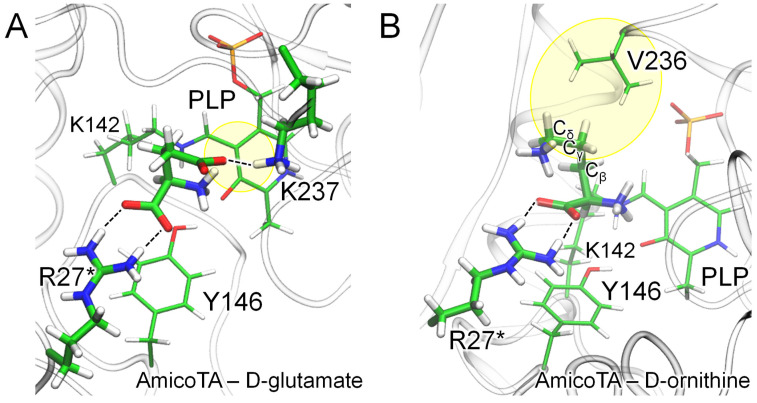
ES complexes of the AmicoTA and D-glutamate (**A**), AmicoTA and D-ornithine (**B**). Hydrogen bonds between the enzyme and the substrate are shown by black dashed lines. Interactions discussed in the text are highlighted in yellow.

We performed MD simulations to examine the substrate binding in the active site of AmicoTA. D-glutamate was found to be the best amino substrate for the AmicoTA, considering both k_max_ and K_D_ values. This is in line with the fact that α-ketoglutarate carrying the similar γ-carboxylate group is the best keto substrate. Classical MD simulations were carried out to obtain the ES complex of WT AmicoTA with D-glutamate (Figure 5A). In the ES complex, the negatively charged α-carboxylate group forms stable salt bridges with the side chain of R27*, and the γ-carboxylate group forms a stable salt bridge with the side chain of K237. We performed additional MD simulations of the ES complexes with other substrates considered in the study. D-aspartate carries a negatively charged β-carboxylate group on its side chain, which does not interact with K237 and is exposed to the solution. This agrees with the almost 60 times increase of the K_D_ value compared with D-glutamate. Unexpectedly, D-ornithine with a protonated amino group in its side chain had a relatively low K_D_ value. MD simulations demonstrate that it forms a stable complex between its hydrophobic part, including C_β_H_2_-C_γ_H_2_-C_δ_H_2_ fragment and a side chain of V236; the protonated amino group is exposed to the solution and shields hydrophobic fragments from interactions with water molecules that should stabilize the Gibbs energy of the entire system (Figure 5B). 

### 2.7. Michaelis Complex of AmicoTA with D-Glutamate and Substrate-Assisted Mechanism of Catalysis

**Figure 6:**
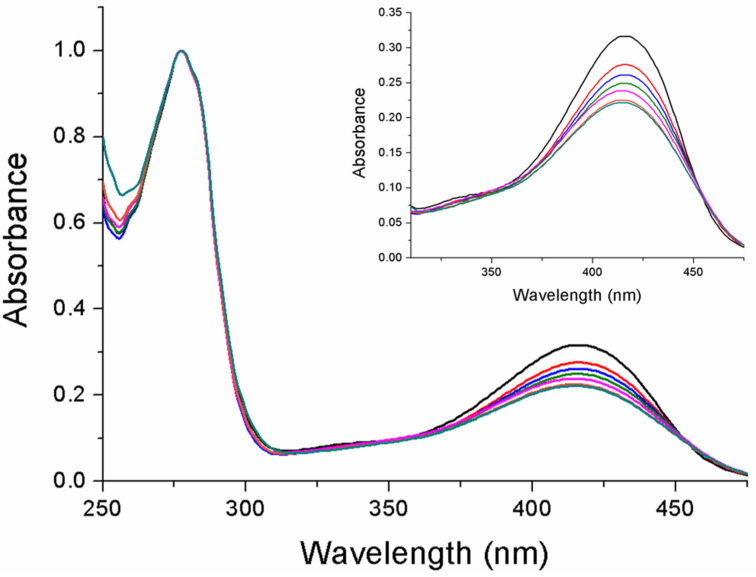
The absorption spectra of the Amico PLP form at a concentration of 30 µM in different buffers: mixed buffer 25 mM Tris-HCl and 25 mM K-phosphate pH 7 (black), pH 8 (red), pH 8.5 (blue), pH 9 (green), 50 mM CHES buffer pH 9 (magenta), pH 9.5 (orange), pH 10 (dark cyan). The insert shows the absorption spectra at 310–465 nm. The spectra were obtained with three independent preparations.

The first process during the transamination reaction is a transimination, that is, the amino group of a substrate substitutes the amino group of catalytic lysine in the internal aldimine, an adduct of PLP and catalytic lysine in the active site of TAs [7,14]. It was shown for TAs of PLP fold type I, that the effectiveness of this step is achieved by the deprotonation of the attacking amino group and concerted protonation of the imine nitrogen atom in the internal aldimine, such combination raises the nucleophilicity of the attacking group and facilitates the leaving group release [7,42]. The absorbance spectra of the holo form of AmicoTA at different pH showed the predominance of the protonated internal aldimine at pH range 7.0–10.0 (Figure 6). No peak at 360–375 nm [32,43] that corresponded to the deprotonated form of the internal aldimine was observed.

**Figure 7:**
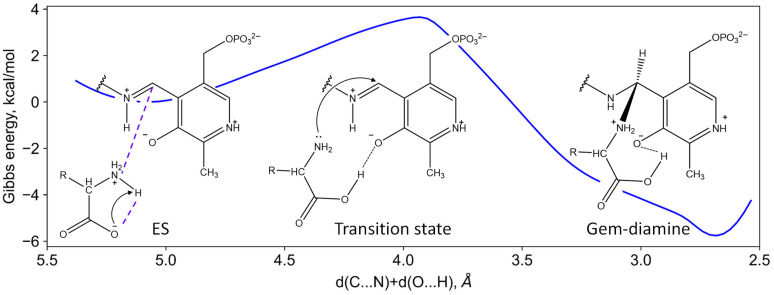
The first step of the reaction leading to the formation of the gem-diamine from the ES complex. Gibbs energy profile is shown in blue, dashed black lines are for hydrogen bonds and dashed violet lines are for the interatomic distances comprising reaction coordinate.

Since the imine nitrogen in the internal aldimine of AmicoTA is protonated, the substrate binding mode should provide an assistance to the deprotonation of the amino group of D-amino acid. No proton acceptor was found in the active site close to the amino group of the substrate except the α-carboxylate of the substrate, which is common for all D-amino acids. Moreover, at optimal pH 8.5 and 9.0, the percentage of the deprotonated form of D-alanine and D-glutamate does not exceed 8% and 25%, correspondingly. Therefore, we examined substrate-assisted catalysis. The mechanism of the first elementary step of the chemical reaction was calculated at the QM(PBE0-D3/6-31G**)/MM MD level of theory. Umbrella sampling procedure was utilized, and the reaction coordinate was chosen as a sum of the distances between the hydrogen atom of the protonated amino group and the oxygen atom of the α-carboxylate group of the substrate, and between the nitrogen atom of the substrate and the C4′ atom of PLP. The Gibbs energy profile is depicted in Figure 7. The substrate acts as a base and the reaction is initiated by the proton transfer from the protonated amino group of the substrate to its α-carboxylate group. In the transition state region, the proton is already transferred to the carboxylate of the α-carboxylate group; the lone pair of the neutral amino group is oriented toward the PLP carbon atom and the distance of the nucleophilic attack is 2.8–2.9 Å that is favorable for the following C—N bond formation. The neutral α-carboxylate group of the substrate is stabilized by the hydrogen bond with the phenyl group of PLP. Thus, at the first step, the gem-diamine is obtained, and the entire system is stabilized by about 6 kcal/mol relative to the ES complex.

## 3. Discussion

AmicoTA is a transaminase of PLP fold type IV, which is active toward D-amino acids and α-keto acids. The highest conversion rate is achieved in transamination between D-alanine and α-ketoglutarate at pH 8.5–9.0, 60 °C. It concedes bsDAAT [34] and Halhy [27] in a catalytic turnover rate in the reaction between D-alanine and α-ketoglutarate 15–25 times in the optimal conditions. At the same time, if we consider *K*m meaning as «the affinity of the enzyme for the substrates in the steady state» [44], these values for α-ketoglutarate are similar in DAATs. It appears that a proper binding of α-ketoglutarate is a key feature of DAATs. In this case, the specificity for other substrates depends on the efficiency of their binding in the active site optimized for α-ketoglutarate. The efficient binding of α-ketoglutarate means the coordination of both carboxylate groups. In AmicoTA, two sites of carboxylate group binding were found: one in the O-pocket formed by R27* and T34, and another in the P-pocket formed by K237 and V236. 

Noteworthy, the O-pocket loop is considered to be a specificity-determining structural element in TAs of fold type IV [9,13,28] because it contains residues for substrate binding. Additionally, the O-pocket loop in closed conformation together with the interdomain loop shields the active site from the solvent. The closed active sites in complexes with substrates are observed for R-TAs and BCATs but not for canonical bsDAAT [10,31,39,40,45,46]. This is not the case for AmicoTA as well. Despite the open active site, in the transamination reaction catalyzed by AmicoTA both the enantiomeric excess and the product yield of the D-amino acids surpass 99% and 98%, correspondingly. These observations support the substrate-differentiation role of the active site closure but not the catalytic one in TAs [9,10,47]. To summarize, the substrate binding in AmicoTA is achieved by multiple interactions with residues located on the fixed structural elements of the active site. The substrate differentiation mechanism should be clarified. Moreover, it remains unclear how DAATs escape the side reaction of racemization.

The mechanism of the overall transamination reaction including the transimination step was established for aspartate aminotransferase from *E.coli* [8,14,48], which demonstrated maximal activity at pH 7.0–8.0 and had deprotonated imine nitrogen at this pH range. In the Michaelis complex, the attacking protonated aspartate donates its proton to imine nitrogen of the internal aldimine, and transimination proceeds [7,14]. The established mechanism of transimination became disputable for DAATs and BCATs [10,22,24,49,50], as the imine nitrogen of the internal aldimine was protonated in the pH optima of these TAs [22,24,49,51]. Goto and coauthors suggested for BCAT from *E. coli* that a proton from the substrate amino group migrated to a negatively charged hole formed in the active site by the phosphate group of cofactor and the adjacent α-carboxylate group of L-amino acid substrate [52]. This hypothesis does not suit for DAATs. In the active site of DAATs, the α-carboxylate group of a substrate and the phosphate group of the cofactor occupy opposite pockets. Here, we suggest substrate-assisted catalysis as a possible route of the amino group deprotonation in a Michaelis complex in the transimination step. The α-carboxylate group of a substrate acts as a proton acceptor, with the Schiff base of the lysine residue and PLP being in a protonated state in the pH range 7–10 and unable to accept a proton. QM/MM MD simulation for AmicoTA demonstrated that the proton transfer from the amino group to the α-carboxylate group in the substrate occurs together with the nucleophilic attack of the substrate nitrogen atom on the internal aldimine carbon atom forming a gem-diamine. This process results in the 6 kcal/mol lowering of the energy of the entire system and happens with the low energy barrier. Our findings are in line with the previous computational study of the related PLP-dependent ornithine decarboxylase [53]. Similar to our findings, the amino group is neutral during the formation of the C-N bond in the gem-diamine and the carboxylate group is neutral as well. The only difference is that in [53], the substrate is already neutral in the Michaelis complex, whereas in our model it is a zwitterion and its transformation to the neutral species occurs in the transition state region of the first elementary step.

## 4. Materials and Methods

### 4.1. Cloning, Expression, and Purification of the Recombinant AmicoTA 

Gene AmicoTA_1844 encoding PLP type IV transaminase (275 a.a., 30.7 kDa) was identified in the genome of *A. colombiense* (strain DSM 12261/ALA-1) [33]. The optimized sequence (http://genomes.urv.es/OPTIMIZER/, accessed on 1 February 2022) was synthesized with 5′ and 3′ overhangs complementing the *NdeI* and *HindIII* restriction sites by ATG Service Gene (St. Petersburg, Russia) and then cloned into the pET-21d vector (Novagen, Darmstadt, Germany) modified as described in [54] to produce a protein fused at the N-terminus with a (His)_6_-tag and a TEV protease site. *E. coli* Rosetta (DE3) pLysS cells (Novagen, Germany) transformed with the construct were grown in LB high salt medium containing 100 µg/mL ampicillin and 34 µg/mL chloramphenicol (Panreac-AppliChem, Darmstadt, Germany) at 37 °C until the OD_600_ value reached 0.8, and then expression was induced with 0.2 mM IPTG. After incubation for 18 h at 30 °C, the cells were harvested by centrifugation, resuspended in a 50 mM K-phosphate buffer, pH 8.0, supplemented with 500 mM NaCl, 20 mM imidazole, 0.5 M urea, 5 mM β-mercaptoethanol, 10% (*v*/*v*) glycerol, 0.2 µg/mL lysozyme, 100 µM PLP, and 1 mM PMSF, and disrupted by sonication. 0.1 mg of DNAse (Sigma-Aldrich, St. Louis, MO, USA) was added to the crude cell extract and it was centrifuged for 45 min at 18,500× *g* at 4 °C. The supernatant was filtered through a 0.45 µm filter (Millipore, Burlington, MA, USA) and applied to a 5 mL HisTrap HP column (Cytiva, Marlborough, MA, USA), equilibrated with 50 mM K-phosphate buffer, pH 8.0, containing 500 mM NaCl, 20 mM imidazole and 0.1% (*v*/*v*) Triton X-100. The (His)_6_-tagged recombinant AmicoTA was eluted with a linear gradient from 20 to 500 mM imidazole in the same buffer without Triton X-100. The target protein was incubated for 1 h with 1 mM PLP at 25 °C, concentrated up to 20–25 mg/mL with a 30 kDa cut-off centrifugal filter device (Millipore, USA) and transferred into storage buffer (50 mM K-phosphate, pH 8.0, containing 100 mM NaCl, 1 mM β-mercaptoethanol and 100 µM PLP), then diluted by glycerol 1:1 and stored at −20 °C. The (His)_6_-tagged recombinant AmicoTA was used for the biochemical characterization.

For crystallization, the fraction of (His)_6_-tagged recombinant AmicoTA was incubated overnight at 4 °C with (His)_6_-tagged TEV protease (1 mg per 10 mg of the protein) solution, containing 1 mM EDTA, 5 mM β-mercaptoethanol and 10% (*v*/*v*) glycerol, then dialyzed against the 50 mM K-phosphate buffer, pH 8.0, containing 500 mM NaCl, 20 mM imidazole and 20 µM PLP, and applied to a HisTrap HP column (Cytiva, USA). A (His)_6_-tagged TEV protease and cleaved (His)_6_-tag were absorbed on the column, whereas the recombinant AmicoTA without (His)_6_-tag was collected in the flow-through mode, concentrated, and applied to a Superdex 200 10/300 GL column (Cytiva, USA) equilibrated in 50 mM HEPES buffer, pH 8.0, containing 100 mM NaCl and 100 µM PLP. The collected fractions were transferred into 20 mM HEPES buffer, pH 8.0, and applied to a MonoQ 10/100 GL column (Cytiva, USA) equilibrated with the same buffer and eluted with a linear NaCl gradient (20–500 mM). The fractions of recombinant AmicoTA were concentrated up to 15–20 mg/mL, transferred to the crystallization buffer: 20 mM HEPES, pH 8.0, supplemented with 50 mM NaCl, 100 μM PLP, and 1 mM DTT, and frozen at −70 °C. Recombinant HGDH from *Acidaminococcus fermentas* was obtained similarly to AmicoTA. The protein purity was analyzed by SDS-PAGE (12%). The protein concentration was determined spectrophotometrically [55]. 

### 4.2. Enzyme Activity Assay

The activity of the purified AmicoTA in the transamination reaction between D-alanine and α-ketoglutarate was determined spectrophotometrically by applying lactate dehydrogenase (LDH) assay on the SPECTROstar Omega (BMG Labtech, GmbH, Ortenberg, Germany) plate reader in the microtiter plates (200 µL) (UV-Star, Greiner Bio-One GmbH -Frickenhausen, Germany). The assay was performed with 5–500 mM D-alanine and 0.5–20 mM α-ketoglutarate, 0.05–0.1 µM of the purified AmicoTA, 330 µM NADH, and 4 U/mL LDH from rabbit muscle (Roche Diagnostics GmbH, Mannheim, Germany) in 50 mM CHES buffer, pH 9.0, containing 60 µM PLP at 60 °C. The reaction was initiated by D-alanine. The reaction progress was monitored by detecting a decrease in the absorbance at 340 nm (ε(NADH) = 6220 M^−1^ cm^−1^). The standard assay was performed with 50 mM D-alanine and 5 mM α-ketoglutarate. The activity of AmicoTA was calculated from the initial linear region of the progress curve of the reaction. One unit (U) was defined as the amount of the enzyme that catalyzed the conversion of 1 µmol of the substrate into a product per minute. 

The activity of the AmicoTA in the transamination reactions between D-glutamate and pyruvate was determined spectrophotometrically by applying (*R*)-2-hydroxyglutarate dehydrogenase (HGDH) assay, respectively, on the SPECTROstar Omega (BMG Labtech, Germany) plate reader in the microtiter plates (200 µL). The HGDH-assay was performed with 1–30 mM D-glutamate and 10–500 mM pyruvate, 0.1–0.3 µM of the enzyme, 330 µM NADH, and 4 U/mL recombinant HGDH from *A. fermentas* in 50 mM K-phosphate buffer, pH 8.0, containing 60 µM PLP at 60 °C. The reaction was initiated by D-glutamate. The reaction progress in HGDH-assay was monitored by detecting a decrease in the absorbance at 370 nm (ε(NADH) = 2484 M^−1^ cm^−1^) because at 340 nm, the absorption from pyruvate at high concentrations was significant. 

Steady-state kinetic parameters of the overall reaction catalyzed by the AmicoTA between D-alanine and α-ketoglutarate, D-glutamate and pyruvate were calculated from the substrate saturation curves at the constant co-substrate concentration using the Michaelis–Menten model.

The kinetic parameters were calculated by fitting the initial velocity data to Equation (1):

(1) $$V=\frac{V_{max}\times A\times B}{K_{m}^{A}\times B+K_{m}^{B}\times A+A\times B}$$

where *V* is the initial velocity, *V_max_* is the maximal velocity, A and B are substrate concentration, and $K_{m}^{A}$ and $K_{m}^{B}$ are the *K*_m_ of substrates *A* and *B*, respectively. All measurements were performed at least in triplicates. The data were analyzed using Origin 8.0 software (OriginLab, Northampton, MA, USA).

### 4.3. Effect of pH and Temperature on the Overall Transamination Reaction

pH and temperature effects were determined in the overall transamination reaction between 50 mM D-alanine and 5 mM α-ketoglutarate and 5 mM D-glutamate and 50 mM pyruvate. The pH optimum was determined at 30 °C using the following buffers: 25 mM Tris-HCl and 25 mM K-phosphate, pH 6–9, and 50 mM CHES buffer, pH 9–10. The temperature dependence of the reaction rate was studied in the range from 30 to 70 °C in 50 mM K-phosphate buffer, pH 8.0. When measuring activity at 65 and 70 °C, the pyruvate and α-ketoglutarate formation was monitored using a discontinuous LDH or HGDH assay, correspondingly, in 100 mM K-phosphate buffer, pH 7.0, at 25 °C [46].

### 4.4. Analysis of Thermal Stability and Operational Stability

The thermal stability of AmicoTA was determined by incubating 45 µM AmicoTA at 40, 50, and 60 °C in 50 mM CHES buffer, pH 9.0, containing 100 µM PLP. The operational stability was determined by incubating 45 µM AmicoTA at 60 °C in 50 mM CHES buffer, pH 9.0, containing 100 µM PLP, 100 mM D-leucine, and 20 mM α-ketoglutarate. Residual activity was measured in the standard assay at several time points for 120 h.

### 4.5. Half-Reaction Assay

The PLP-form of AmicoTA was obtained by incubation with an excess of both PLP and α-ketoglutarate for 1 h at 25 °C, followed by transfer into 50 mM CHES buffer, pH 9.0, using a 5 mL Desalting column (Cytiva, USA). Half-transamination reactions of the AmicoTA PLP form (35–40 µM) with D-amino acids were followed spectrophotometrically at 408 nm by measuring a decrease in the aldimine concentration in presence of different concentrations of D-amino acids in 50 mM CHES buffer, pH 9.0 at 40 °C in UV-transparent microtiter plates (UV-Star, Greiner, Germany) using SPECTROstar Omega plate reader (BMG Labtech, Germany). The rate constants of half-reactions were determined by fitting Equation (2):

(2) $$A_{t}=A_{\infty }+\Delta Aexp\left(-k_{\mathrm{obs}}t\right)$$

where $A_{t}$ is the absorbance at time *t*, ΔA is the difference between absorbance at *t* = 0 and *t* = ∞, $A_{\infty }$ is the final absorbance, and $k_{\mathrm{obs}}$ is the observed rate constant. The dissociation constant for the enzyme–substrate complex $K_{D}$, the maximal rate constant $k_{\max}$, the rate constant of the reverse reaction $k_{r}$, and the specificity constant kmaxKD were obtained by fitting Equation (3):

(3) $$k_{\mathrm{obs}}=\frac{k_{\max}\left[S\right]}{K_{D}+\left[S\right]}+k_{r}$$

All measurements were performed at least in triplicates. The data were analyzed using Origin 8.0 software.

### 4.6. Analysis of the Product Yield and Enantiomeric Excess in the Transamination Reaction

The product yields were determined in the catalyzed by AmicoTA reactions between *4-methyl-2-oxovalerate* and *D-glutamate* and between *phenylpyruvate* and *D-glutamate* by determining the consumption of 4-methyl-2-oxovalerate or the formation of D-phenylalanine. A one-pot three-enzyme system was employed to shift the equilibrium toward the products. The coproduct, α-ketoglutarate, was removed from the reaction mixture by HGDG assay while recovering NADH in D-glucose conversion catalyzed by glucose dehydrogenase. The reaction mixture contained 100 mM K-phosphate buffer, pH 7.5, 100 µM PLP, 100 mM D-glutamate, 50 mM 4-methyl-2-oxovalerate or 50 mM phenylpyruvate, 4 mg/mL WT AmicoTA, 1 mM NADH, 150 mM D-glucose, 180 U/mL HGDH, and 30 U/mL glucose dehydrogenase (Sigma, USA). The reaction mixtures were incubated at 30 °C with 4-methyl-2-oxovalerate for 20 h and with phenylpyruvate for 40 h. The reactions were terminated by removing the enzyme using an Amicon-Ultra-15 centrifugal tube (Millipore, USA), and then the filtrate was analyzed by HPLC (ÄKTA Purifier, Marlborough, MA, Cytiva, USA) using a reverse-phase C18 column (Zorbax Eclipse XDB-C18, 5 µm, 4.6 × 150 mm, (Agilent, Santa Clara, CA, USA)). The chiral analysis of produced D-leucine and D-phenylalanine was performed by HPLC using a reverse-phase C18 column with a UV detector set at 340 nm. HPLC conditions are described in the Supplementary Materials.

### 4.7. Crystallization and Data Collection

Initial crystallization screening was performed on a robotic system (Rigaku Americas Corporation, The Woodlands, TX USA) using 96-well VDX plates (Hampton Research, Aliso Viejo, CA USA) and commercial crystallization screens from Hampton Research (Aliso Viejo, CA USA) and Molecular Dimensions Inc (Holland, OH USA) by the “hanging drop” vapor diffusion method. A 15 mg/mL of the AmicoTA holo form in 20 mM HEPES buffer pH 8.0 containing 50 mM NaCl, 100 μM PLP, and 1 mM DTT was mixed with the crystallization solution in the ratios 1:1, 1:2 (0.1 μL drop volume), and 2:1 (0.2 μL drop volume). The volume of the precipitant solution in the reservoir was 50 µL. The initial crystallization hit was observed under the following conditions: 0.2 M sodium nitrate, 0.1 M Bis-tris propane, pH 6.5, 20% PEG3350 at 1:1 ratio at 288 K. Further optimization of crystal growth was made using the “hanging drop” vapor-diffusion method in 24-well VDX plates (Hampton Research). The drop volume was increased to 3 μL, and the volume of the precipitant solution to 500 μL. 

Crystals of the AmicoTA complex with D-glutamic acid were obtained by soaking the crystal of the holoenzyme in a crystallization solution containing 100 mM substrate for 5 min.

Crystals of the AmicoTA holoenzyme and its complex were briefly soaked in a mother liquor containing 20% glycerol immediately before diffraction data collection and flash-frozen in liquid nitrogen. Datasets were collected at 100K at ID23-1 beamline (ESRF, France) [56] and Rigaku OD XtaLAB Synergy-S (IOC RAS, Russia) for holoenzyme and complex with D-glutamate, respectively. The datasets for holoenzyme were indexed, integrated, and scaled using the XDS package [57], while for the complex with D-glutamate the CrysAlisPro software v.1.0.43 (Oxford Diffraction/Agilent Technologies UK Ltd., Yarnton, UK) was used. Space groups were suggested by Pointless [58] as P2_1_2_1_2_1_ for both structures (Table 5).

### 4.8. Structure Solution and Refinement

The structure of the holo form of AmicoTA was solved by the molecular replacement method using the MOLREP program [59] with the atomic coordinates of the BCAT transaminase from the archaeon *Geoglobus acetivorans* (PDB ID: 5E25) as a starting model, while the structure of the AmicoTA complex was solved using the AmicoTA holo form structure. Two copies of the protein were found in an asymmetric unit of both structures.

The refinement of all structures was carried out using the REFMAC5 program of the CCP4 suite [60]. The visual inspection of electron density maps and the manual rebuilding of the model were carried out using the COOT interactive graphics program [61]. The isotropic B-factor and the hydrogen atoms in fixed positions were included during the refinement. In both final models, the protein subunits within the asymmetric unit have a similar fold with corresponding pairwise RMSD not exceeding 0.25Å.

### 4.9. Structure Analysis and Validation

The visual inspection of the modeled structure was carried out using the COOT program and the PyMOL Molecular Graphics System, Version 4.6 (Schrödinger, USA). The structure comparison and superposition were made using the PDBeFOLD program [62]. The contacts were analyzed using the PDBePISA [63]. 

### 4.10. Molecular Modeling

The crystal structure of the AmicoTA in the PLP bound form obtained in this study was utilized as a source of coordinates of heavy atoms. Substrates were incorporated in the active sites of both monomers of the dimeric structure. We studied complexes with D-glutamate, D-aspartate, D-ornithine, and D-alanine. Simulations for all model systems were performed mimicking pH9; aspartate and glutamate residues were negatively charged, lysine and arginine residues were protonated and histidine residues were neutral. The protonation state of either the Nδ or Nε atom of the histidine side chain was chosen depending on its local environment. All MD simulations were performed in NAMD3 [64] with the 1 fs integration time step at p = 1 atm and T = 300 K with the length of each trajectory 200–300 ns. CHARMM36 parameters [65] were utilized for protein, TIP3P [66] for water molecules and modified CGenFF [67] for the PLP. 

QM/MM MD simulations were performed in the TeraChem [68] and NAMD2 programs [69] using a specific interface [70]. QM subsystem was described at the PBE0-D3/6-31G** level [71]; it included the PLP and the part of the K142, D-glutamate, and neighboring residues that form hydrogen bonds. Umbrella sampling simulations were performed for the first step of a chemical reaction. Different reaction coordinates were tested during the simulations. First, we examined the possible reaction path as a set of sequential processes: a proton transfer followed by the nucleophilic attack as two separate steps. To do this, the reaction coordinate for the first step was suggested as a difference between two distances along the proton transfer, N…H and O…H distances. We failed to locate a minimum corresponding to the neutral substrate; therefore, we next tried to use a reaction coordinate that comprised both proton transfer and nucleophilic attack. The reaction coordinate was set as a sum of the distances between the hydrogen atom of the protonated amino group and an oxygen atom the α-carboxylate group of the substrate and between the nitrogen atom of the substrate and a C4′ atom of PLP. A set of 10 ps QM/MM MD simulations were performed with harmonic potentials added on reaction coordinate with the force constant of kcal/mol/Å^2^ and centered from 2.7 Å to 5.1 Å with 0.3 Å step. The transition state region corresponds to the reaction coordinate around 3.9 Å and the corresponding QM/MM MD trajectory is available at ZENODO (https://doi.org/10.5281/zenodo.7633915, accessed on 21 February 2023). Umbrella integration was applied to reconstruct the Gibbs energy profile. 

## 5. Conclusions

D-amino acid transaminase from *A. colombiense* (AmicoTA) has been identified and characterized. The enzyme is active toward various D-amino acids and α-keto acids and can be applied for stereoselective amination of compounds with α-carboxylate group at pH 7–10 in the temperature range 30–60 °C. The active site of AmicoTA is set up for a multipoint binding of α-ketoglutarate or D-glutamate. The binding of other substrates is less effective. The substrate binding mode does not include residues from the O-pocket loop, the multipoint substrate binding is provided by the residues from the fixed structural elements of the active site. The same binding mode seems to be realized in the homologous transaminases from *C. pusillum*, *H. hydrossis*, *M. tuberculosis*, and *A. thaliana*. To the best of our knowledge, in the studied transaminases of PLP fold type IV, the residues of mobile O-pocket loop typically participate in the binding of substrates, thus providing the effective catalysis of structurally different substrates. Obviously, in AmicoTA and homologous DAATs, the plasticity of the active site is achieved differently. The study of substrate binding and substrate differentiation mechanism in non-canonical DAATs will be continued.

## Figures and Tables

**Table 1:** Structure-based sequence alignment of AmicoTA and known DAATs. Amino acid composition of SSE forming the active sites of DAATs. Crystal structures of the following DAATs were analyzed: DAAT from *Bacillus* sp. YM-1(PDB ID 1DAA), *B. thailandensis* (4TM5), *C. pusillum* (5K3W), *H. hydrossis* (7P7X), and *M. tuberculosis* (6Q1R). AlphaFold models (accessed on 21 February 2023) [35,36] of the following DAATs were analyzed: DAAT from *B. sphaericus* (UniProt ID P54693), *G. toebii* SK1 (Q4JFX0), *L. salivarius* (Q1WRM6), and *A. thaliana* (Q8L493). Amino acid residues known to participate in substrate binding are shown in red. Similar residues in homologs are shown in bold. Positively charged residues in the P-pockets are shown in bold and *italics*. Canonical DAATs are highlighted in rose, non-canonical DAATs are shown in gray.

DAAT from	Identity, %	O-Pocket α-Helix	βX-Strand	βY-Strand	O-Pocket Loop	Interdomain Loop	β-turn1	β-turn2
*Bacillus* sp. YM-1	100	^21^DRGYG**F**G^27^	^29^GV**Y**EVV***K***VY^37^	^85^GHI**Y**FQVT^92^	^93^RGTSP**R**A**H**QFPENTVKP^109^	^117^NPRPLENLEKG^128^	^178^GSSS^181^	^240^STTS^243^
*B. sphaericus*	68.0	^22^DRGYQ**F**G^28^	^30^GI**Y**EVI***K***VY^38^	^86^GHV**Y**FQIT^93^	^94^RGTTS**R**N**H**IFPDASVPA^110^	^119^GERSIEQFEKG^129^	^179^CSSA^182^	^241^SVSS^244^
*B. thailandensis*	37.5	^32^DRGFI**F**G^38^	^40^GV**Y**EVV**P**IY^48^	^05^AIV**Y**IQVT^112^	^113^RGVAK**R**G**H**AFPANAVP^128^	^137^LALPTDAQRAQG^147^	^198^GSSS^201^	^260^SAT***K***^263^
*G. toebii* SK1	48.0	^25^ERGLQ**F**G^31^	^33^GV**Y**EVA***R***IY^41^	^90^AIL**Y**LQVT^97^	^98^RGSFP**R**N**H**AFPAENRP^113^	^122^MPRKIREIEQG^132^	^182^GSSS^185^	^244^STTS^247^
*L. salivarius*	35.9	^23^DRALY**F**G^29^	^31^GC**Y**DAT**T**FK^39^	^87^GIL**Y**WQTS^94^	^95^RGSGL**R**N**H**IFPEDSQP^110^	^118^PYGLVPFDTE^127^	^177^CAHS^180^	^240^SSAC^242^
*C. pusillum*	23.1	^46^DLGIT**R**G^52^	^54^GV**F**ETI**A**VI^62^	^114^LFA**K**LILT^121^	^122^RGIEGEGRP^130^	^139^GEDFSQQRLG^148^	^208^GPTS^211^	^270^SSV***R***^273^
*H. hydrossis*	25.9	^23^DLSIL**R**G^29^	^31^GI**F**DYF**L**AR^39^	^87^AGI**R**LVLT^94^	^95^GGYSPDGYTVNP^107^	^115^DLPASAWEFSAQG^127^	^177^SARS^180^	^238^STI***K***^241^
*M. tuberculosis*	23.6	^21^DLAAV**R**G^27^	^29^GV**F**ETL**L**VR^37^	^7^GAL**R**LIYS^94^	^95^RGREGGSAP^103^	^112^VPARVIGARRDG^123^	^183^GPRS^186^	^252^SSMT^255^
*A. thaliana*	23.8	^99^DHMVH**R**G^105^	^107^GV**F**DTA**L**II^115^	^163^GSL**R**YWLS^170^	^171^AGPGDFLLSPSQCLLP^186^	^195^TNFAINPIG^203^	^256^GPNM^259^	^324^GSGI^327^
AmicoTA	28.7	^22^DLIIQ**R**G^28^	^30^GV**F**ETI**S**TH^38^	^85^TMV**R**PYIT^92^	^93^GDSFG**K**D**H**LFSSSRYFV^110^	^115^IRKPDPILYEKG^126^	^173^GSHS^176^	^234^GTV***K***^237^

**Table 2:** The specificity-determining motifs of two groups of DAATs. Numeration as in bsDAAT (PDB ID 1DAA).

Group	Sequence Motif 1				Sequence Motif 2	
	26	31	33	35	x88xx	98xx
Canonical DAATs	F	Y	[VA]	[KRPT]	xYxQ	RxH
Non-canonical DAATs	R	F	[TY]	[LA]	x[RK]x[IVW]	Non-conservative

**Table 3:** Kinetic parameters of half-reactions between the PLP form of AmicoTA and amino donors in 50 mM CHES buffer, pH 9.0, at 40 °C. ND—not detected. The values of the rate constants of the reverse reactions were zero for all substrates.

Amino Donor	10^3^ × k_max_, s^−1^	K_D_, mM	k_max_/K_D_, M^−1^ s^−1^
D-Glutamate	1080 ± 40	2.1 ± 0.2	515 ± 70
D-Alanine	280 ± 10	50 ± 6	5.6 ± 0.8
D-Aspartate	118 ± 6	120 ± 15	1.0 ± 0.2
D-Leucine	33 ± 2	100 ± 10	0.33 ± 0.05
D-Ornithine	2.1 ± 0.1	10 ± 1	0.21 ± 0.02
D-Phenylalanine	2.9 ± 0.2	19 ± 3	0.15 ± 0.03
L-Alanine	ND		
L-Leucine	ND		
(*R*)-(+)-1-phenylethylamine	ND		
(*S*)-(−)-1-phenylethylamine	ND		

**Table 4:** Kinetic parameters of the overall transamination reactions between D-alanine and α-ketoglutarate (in 50 mM CHES buffer, pH 9.0) and the reverse reaction between D-glutamate and pyruvate (in 50 mM K-phosphate buffer, pH 8.0), catalyzed by AmicoTA, at 60 °C.

Substrate	Co-Substrate	*k_cat_*, s^−1^	*K*_m_, mM	*k_cat_*/*K*_m_, M^−1^ s^−1^
D-alanine	5 mM α-ketoglutarate	9.9 ± 1.3	465 ± 60	22 ± 6
α-ketoglutarate	500 mM D-alanine	9.9 ± 1.3	7.2 ± 0.8	1375 ± 330
D-glutamate	500 mM pyruvate	1.40 ± 0.06	5.2 ± 0.6	270 ± 40
pyruvate	30 mM D-glutamate	1.40 ± 0.06	160 ± 20	9 ± 1

**Table 5:** Data collection and refinement statistics.

	*Holo form of AmicoTA*	*The Complex of AmicoTA with D-Glutamate*
**Diffraction source**	ESRF (ID23-1 beamline)	Institute of Organic Chemistry RAS (Rigaku OD XtaLAB Synergy-S)
**Wavelength (Å)**	0.98	1.54
**Temperature (K)**	100	
**Detector**	PILATUS 6M	HyPix-6000HE
**Crystal-to-detector distance (mm)**	250	33
**Rotation range per image (°)**	0.05	0.3
**Total rotation range (°)**	120	300
**Space group**	P2_1_2_1_2_1_	P2_1_2_1_2_1_
***a*, *b*, *c* (Å)**	61.19, 80.97, 98.96	61.55, 90.01, 100.86
**α, β, γ (°)**	90.0, 90.0, 90.0	90.0, 90.0, 90.0
**Average mosaicity (°)**	0.273	1.04
**Resolution range (Å)**	43.78–1.90 (1.94–1.90)	21.48–1.90 (1.94–1.90)
**Completeness (%)**	93.0 (92.5)	98.8 (97.5)
**Average redundancy**	4.5 (4.4)	10.8 (11.3)
**〈*I*/σ(*I*)〉**	10.1 (1.5)	19.3 (2.6)
**Rmeas (%)**	8.9 (100.4)	11.1 (116.8)
**CC_1/2_**	99.8 (55.8)	99.9 (83.1)
***R_fact_* (%)**	18.9	19.5
***R*_free._ (%)**	24.5	21.1
**Bonds (Å)**	0.02	0.01
**Angles (°)**	1.98	1.66
**Most favored (%)**	98.5	98.7
**Allowed (%)**	1.5	1.3
**PDB entry code**	8AHR	8AYK

## Data Availability

The data presented in this study are available on request from the corresponding author.

## Supplementary Materials

The following supporting information can be downloaded at: https://www.mdpi.com/article/10.3390/molecules28052109/s1, Table S1: Superposition of the AmicoTA subunit with the homologous TA of PLP fold type IV; Figure S1: Structure-based sequence alignment of AmicoTA and known DAATs. Figure S2: SDS-PAGE of fractions of AmicoTA when expressed in *E. coli* and purified. Figure S3: Half-reactions catalyzed by AmicoTA. Figure S4: Concentration dependences of the specific activity of AmicoTA in the overall transamination reactions. Figure S5: Thermal stability and operational stability of the PLP form of AmicoTA. Figure S6: HPLC analysis of the configuration of the products of the overall transamination reaction catalyzed by AmicoTA. Figure S7: The gel filtration elution profile for Amico TA; Figure S8: Binding of PLP molecule in the holoenzyme.

## Sample Availability

Samples of AmicoTA are available from the author, other compounds can be ordered in Sigma.
