# Supplementary material for: To the Understanding of Catalysis by D-Amino Acid Transaminases: A Case Study of the Enzyme from Aminobacterium colombiense
Source: Molecules. 2023 Feb 23;28(5):2109. doi: 10.3390/molecules28052109 (PMC10003956; doi:10.3390/molecules28052109)
Supplement: Supplementary file 1 [file molecules-28-02109-s001.zip › molecules-2221115-supplementary.pdf]

Molecules

Supplementary

To the understanding of catalysis by D-amino acid transaminases: a case study of the enzyme from *Aminobacterium colombiense*

Sofia A. Shilova[1#], Maria G. Khrenova [1,2#], Ilya O. Matyuta[1], Alena Y. Nikolaeva[1,3] , Tatiana V. Rakitina[1,4], Natalia L. Klyachko[2], Mikhail E. Minyaev[5], Konstantin M. Boyko[1], Vladimir O. Popov[1,6], and Ekaterina Yu. Bezsudnova [1,*]

[1]Bach Institute of Biochemistry, Research Centre of Biotechnology of the Russian Academy of Sciences, 119071, Moscow, Russia
[2]Department of Chemistry, Lomonosov Moscow State University, 119991, Moscow, Russia
[3]Complex of NBICS Technologies, National Research Center "Kurchatov Institute", 123098, Moscow, Russia;
[4]Shemyakin-Ovchinnikov Institute of Bioorganic Chemistry of the Russian Academy of Sciences, 117997, Moscow, Russia
[5]N.D. Zelinsky Institute of Organic Chemistry, Russian Academy of Sciences, 119991, Moscow, Russia
[6]Department of Biology, Lomonosov Moscow State University, 119991, Moscow, Russia

# - These authors contributed equally to this work.

* Correspondence: eubez@inbi.ras.ru;

Content:

Tables S1 ……………………………………………………………………..…p. 2

Figures S1………………………………………………………………….…p. 3

Figures S2………………………………………………………….….......p. 4

Figures S3-S4……………………………………………………………pp. 5-7

Figures S5………………………………………………………….….......p. 8

Figure S6. Product yield and enantiomeric excess……………………….….pp. 9-10

Figure S7-S8…………………………...………………………………………..p. 11

Table S1. Superposition of the AmicoTA subunit with the subunits of homologous TAs of PLP fold type IV.

TA from (PDB ID)	Type	RMSD, Å	Z-score	Sequence identity, %	Percentage of the Aligned Residues, %
Archaeoglobus fulgidus (5MR0)	BCAT	1.5	14.7	35	89
Geoglobus acetivorans (5E25)		1.6	14.6	32	84
Escherichia coli (1I1L)		1.8	11.7	27	68
Haliscomenobacter hydrossis (7P7X)	DAAT	1.6	13.5	30	89
Bacillus subtilis (1DAA)		1.8	12.5	26	84
Burkholderia thailandensis (4TM5)		1.8	12.9	27	84
Aspergillus terrus (4CE5)	R-TA	1.7	14.0	21	100
Aspergillus fumigatus (4CHI)		1.6	13.2	20	74
Exophiala xenobiotica (6FTE)		1.7	12.6	22	95
Curtobacterium pusillum (5K3W)	TA*	2.0	11.4	24	84
Haliangium ochraceum (6H65)		1.9	11.9	23	89

*with a broad substrate specificity.

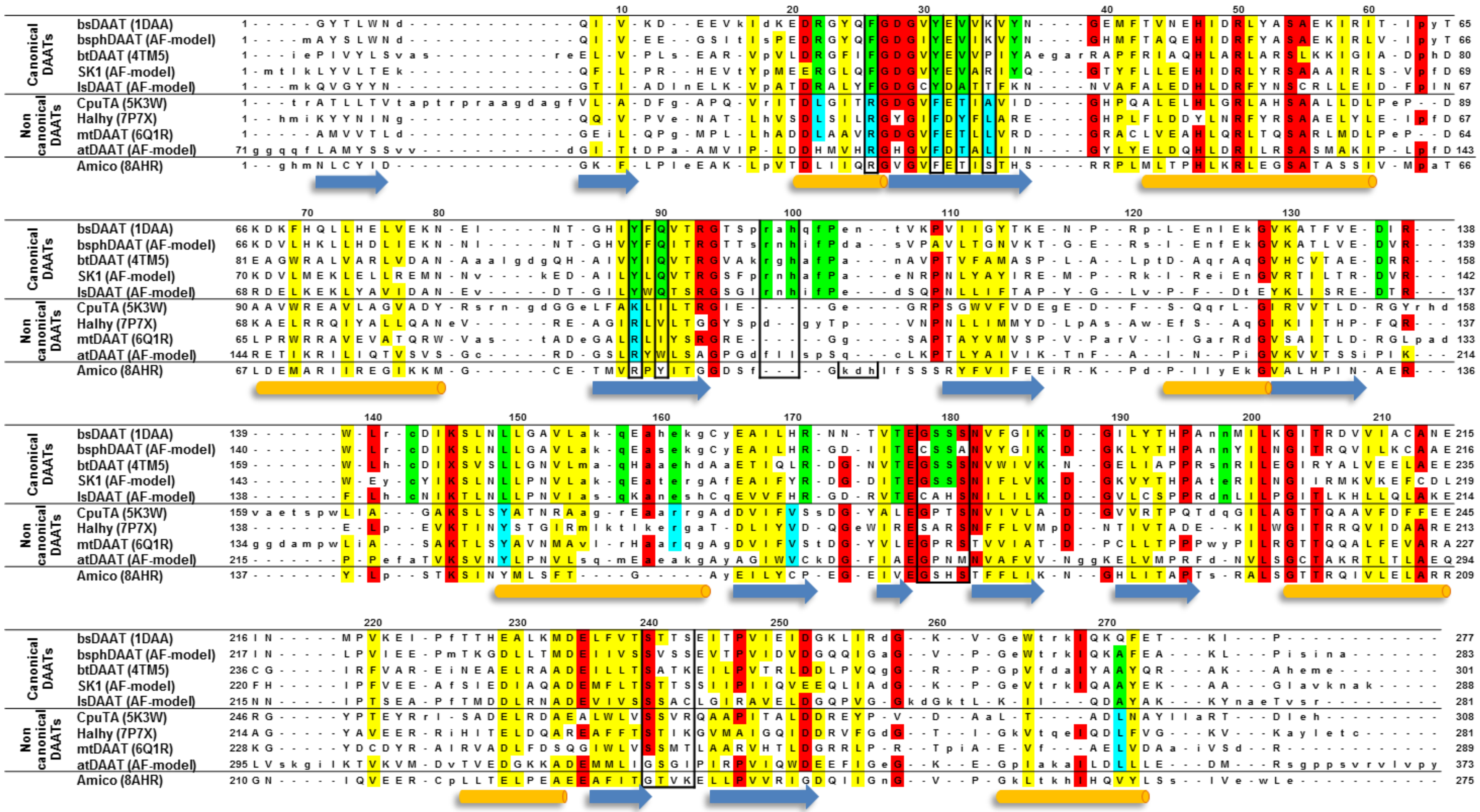

Figure S1. Structure-based sequence alignment of AmicoTA and known DAATs. Crystal structures of the following DAATs were used: DAAT from *Bacillus* sp. YM-1 (bsDAAT, PDB ID 1DAA), *Burkholderia thailandensis* (btDAAT, PDB ID 4TM5), *Curtobacterium pusillum* (CpuTA, PDB ID 5K3W), *Haliscomenbacter Hydrossis* (Halhy, PDB ID 7P7X) and *Mycobacterium tuberculosis* (mtDAAT, PDB ID 6Q1R). AlphaFold models (https://alphafold.ebi.ac.uk/) of following DAATs were used: DAAT from *Bacillus sphaericus* (bsphDAAT, UNIPROT ID P54693), *Geobacillus toebii* SK1 (SK1DAAT, Q4JFX0), *Lactobacillus salivarius* (lsDAAT, Q1WRM6) and *Arabidopsis thaliana* (atDAAT, Q8L493). Identical residues in all DAATs are in red, similar residues are in yellow, identical residues in ONLY canonical or non-canonical DAATs are in green and cyan, respectively. β-strands are shown as blue arrows and α-helixes – as orange cylinders. The residues forming the active site are shown in frames.

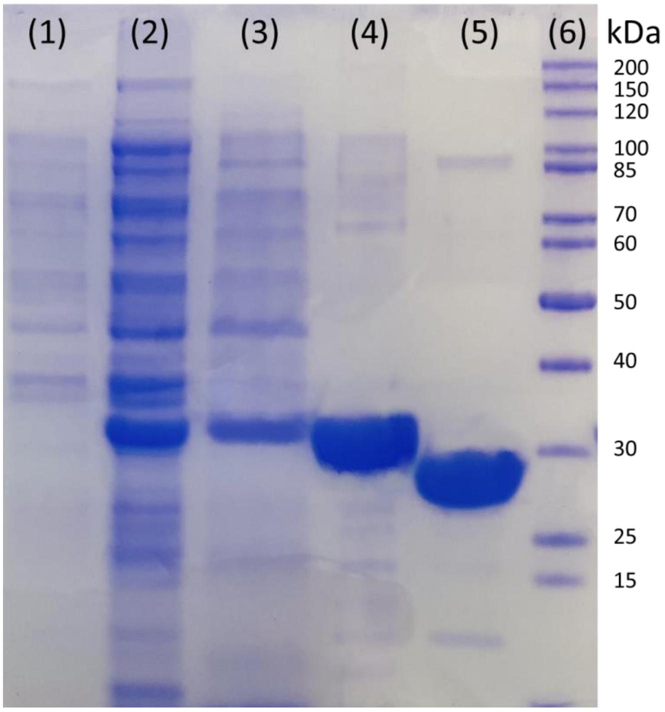

Figure S2. SDS-PAGE of fractions of AmicoTA when expressed in *E. coli* and purified. (1) Cells lysate before induction; (2) cells lysate after IPTG induction; (3) cells lysate after sonication and centrifugation; (4) fraction of AmicoTA-His$_6$TEVtag after HisTrap HP column; (5) fraction of AmicoTA after cleavage of His$_6$-tag, using TEV-protease, gel-filtration and anion exchange chromatography; (6) Page Ruler Unstained Protein Ladder (Thermo Fisher Scientific, Waltham, MA, USA).

Figure S3. Half-reactions catalyzed by AmicoTA

1. D-Glutamate

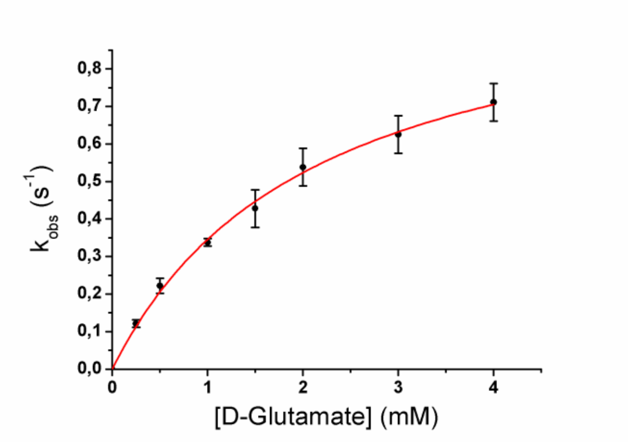

$k_{max} = 1.08 \pm 0.04\ s^{-1}$

$K_D = 2.1 \pm 0.2$ mM

kr = 0

2. D-Alanine

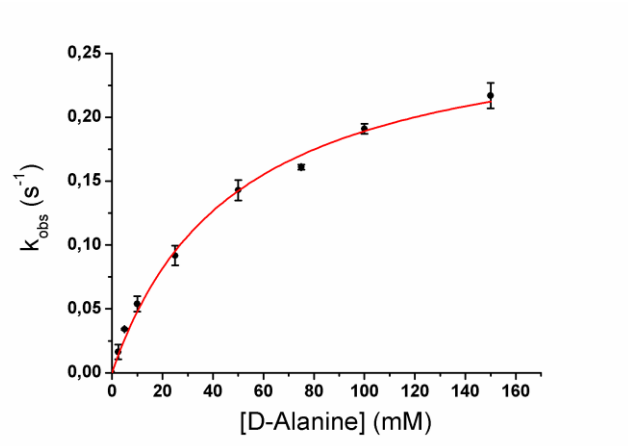

$k_{max} = 0.28 \pm 0.01\ s^{-1}$

$K_D = 50 \pm 5.5$ mM

kr = 0

3. D-Aspartate

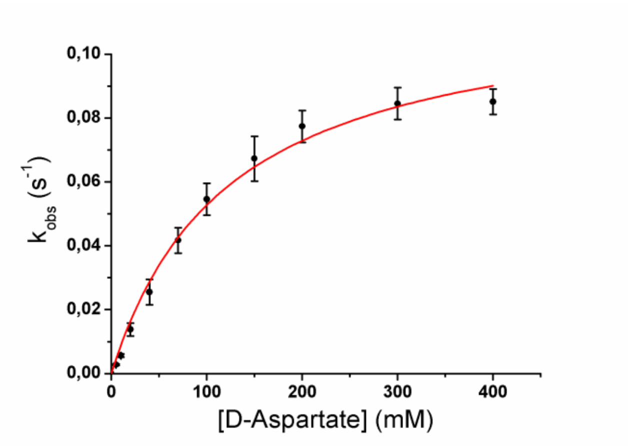

$k_{max} = 0.118 \pm 0.006\ s^{-1}$

$K_D = 120 \pm 15$ mM

kr = 0

4. D-Leucine

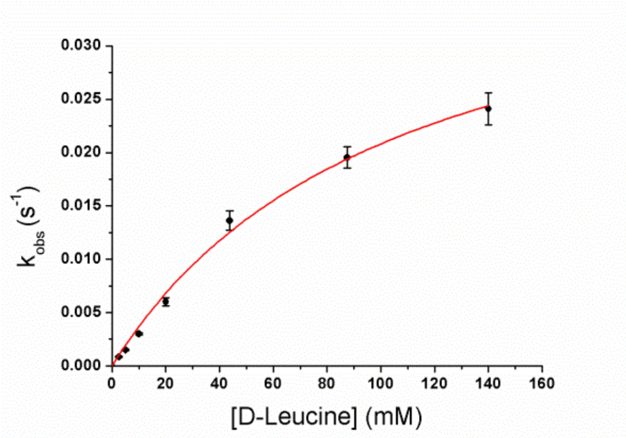

$k_{max} = 0.033 \pm 0.002\ s^{-1}$

$K_D = 100 \pm 10$ mM

kr = 0

5. D-Ornithine

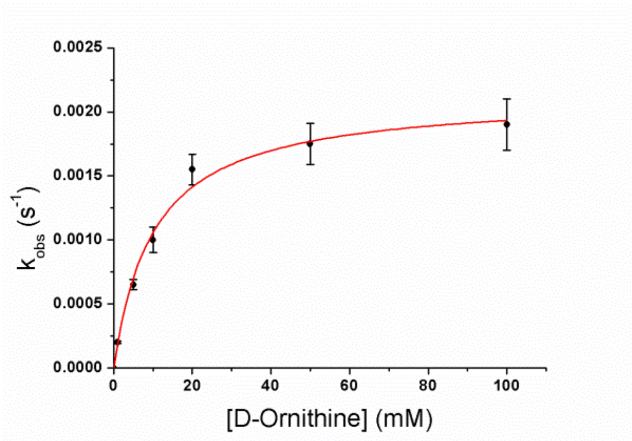

$k_{max} = 0.0021 \pm 0.0001\ s^{-1}$

$K_D = 10 \pm 1$ mM

kr = 0

6. D-Phenylalanine

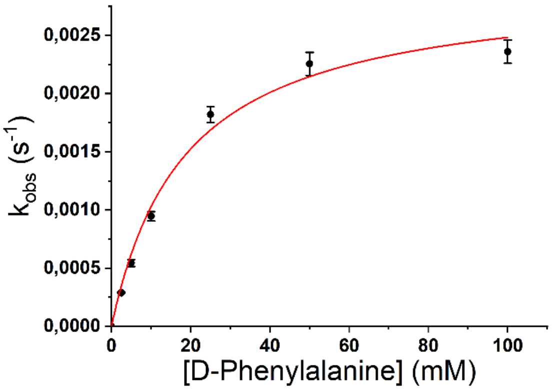

$k_{max} = 0.0029 \pm 0.0002\ s^{-1}$

$K_D = 19 \pm 3$ mM

kr = 0

Figure S4. Concentration dependences of the specific activity of AmicoTA in the overall transamination reactions.

D-Alanine + a-Ketoglutarate

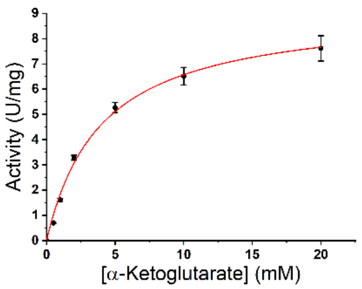

[D-Alanine]$_{fix}$ = 500 mM

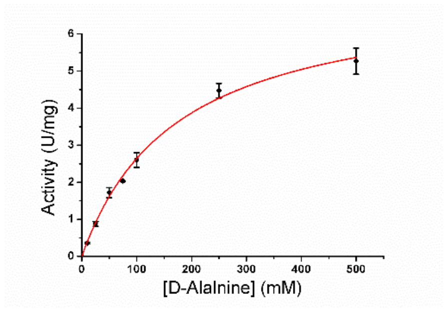

[α-Ketoglutarate]$_{fix}$ = 5 mM

$$V_m^{app} = 9.2 \pm 0.3 \frac{U}{mg}$$
$$K_M^{app}(\alpha - KG) = 4.0 \pm 0.4\, mM$$

$$V_m^{app} = 7.2 \pm 0.3 \frac{U}{mg}$$
$$K_M^{app}(D - Ala) = 175 \pm 15\, mM$$

$$V_m = 17.9 \pm 2.4 \frac{U}{mg}$$
$$k_{cat} = 9.9 \pm 1.3\, s^{-1}$$
$$K_M(\alpha - KG) = 7.2 \pm 0.8\, mM$$
$$K_M(D - Ala) = 460 \pm 60\, mM$$

D-Glutamate + Pyruvate

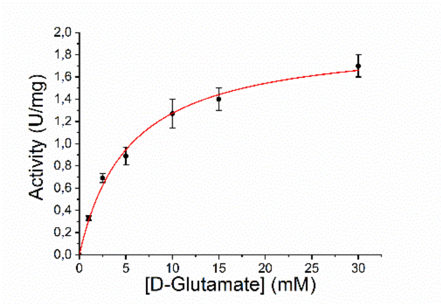

[Pyruvate]$_{fix}$ = 500 mM

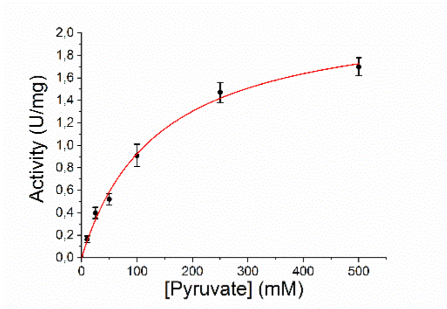

[D-Glutamate]$_{fix}$ = 30 mM

$$V_m^{app} = 1.95 \pm 0.07 \frac{U}{mg}$$
$$K_M^{app}(D - Glu) = 5.3 \pm 0.6\, mM$$

$$V_m^{app} = 2.2 \pm 0.1 \frac{U}{mg}$$
$$K_M^{app}(Pyr) = 140 \pm 15\, mM$$

$$V_m = 2.5 \pm 0.1 \frac{U}{mg}$$
$$k_{cat} = 1.40 \pm 0.06\, c^{-1}$$
$$K_M(D - Glu) = 5.2 \pm 0.6\, mM$$
$$K_M(Pyr) = 160 \pm 20\, mM$$

Figure S5. Thermal stability (A) and operational stability (B) of the PLP form of AmicoTA. Temperature 40 °C is black, 50 °C – red and 60 °C - green. Thermal stability was measured by incubating 45 µM AmicoTA in PLP form in 50 mM CHES buffer, pH 9.0, with 100 µM PLP. Operational stability was measured by incubating 45 µM AmicoTA in PLP form in 50 mM CHES buffer, pH 9.0, contained 100 µM PLP, 100 mM D-leucine and 20 mM α-ketoglutarate. 100% corresponds to 1.5 ± 0.1 U/mg in the standard assay.

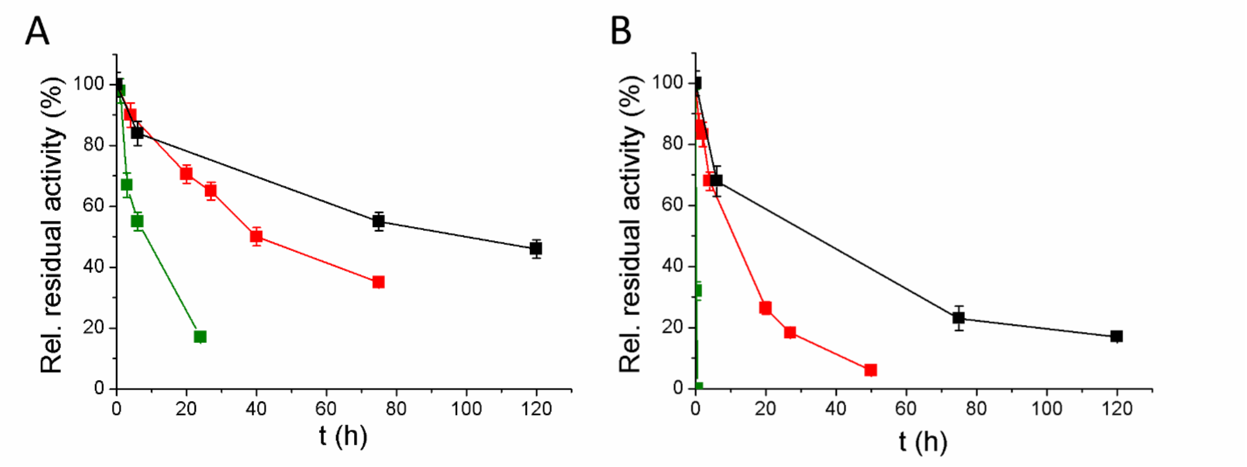

Figure S6. Determination of the product yield and enantiomeric excess in the reactions between D-glutamate and 4-methyl-2-oxovalerate and between D-glutamate and phenylpyruvate catalyzed by AmicoTA.

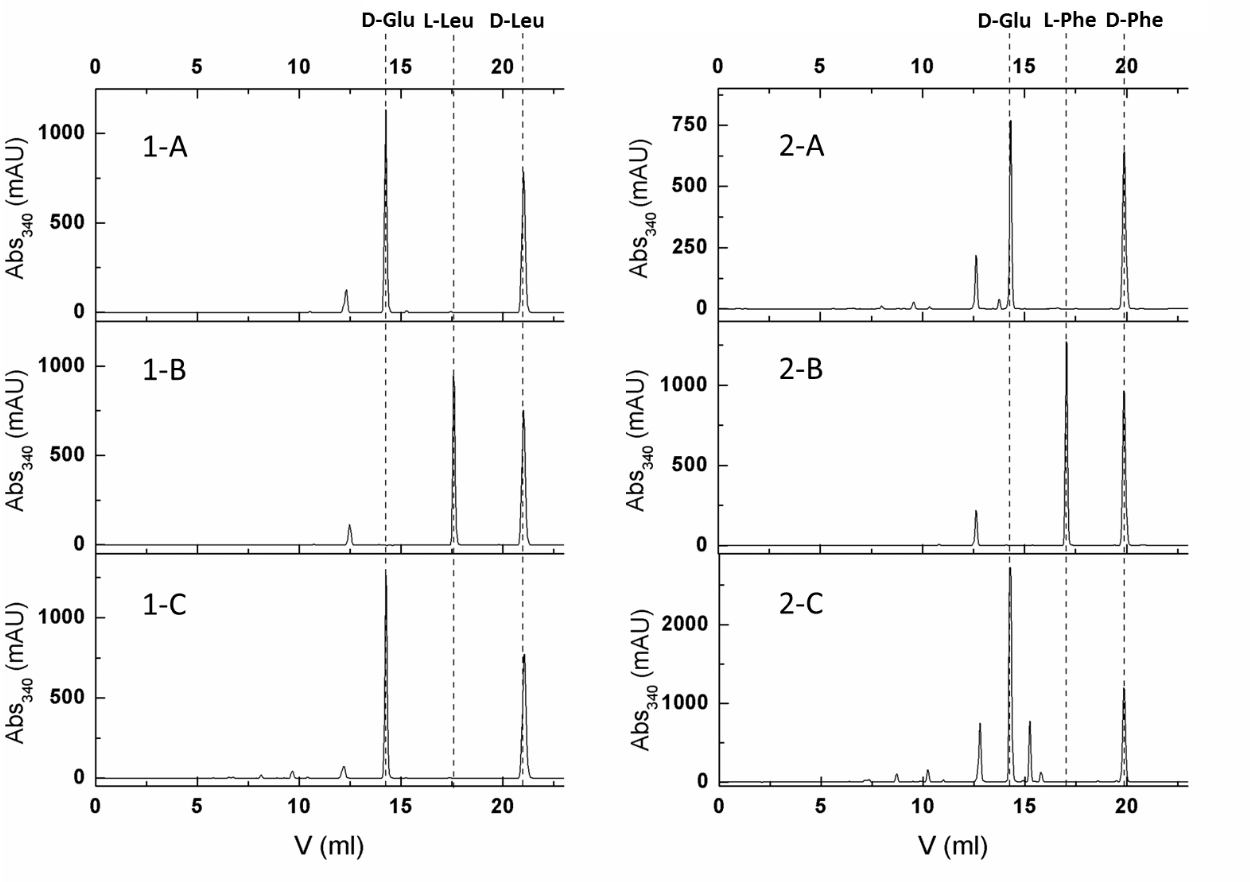

HPLC analysis of the configuration of the products of the overall transamination reaction catalyzed by AmicoTA. Chromatograms of standards and samples (leucine, phenylalanine and glutamate) derivatized with Marfey's reagent. 1-A: reference standards of D-glutamate and D-Leu at concentration of 50 mM; 1-B: reference standards of D- and L-leucine at concentration of 50 mM; 1-C: the reaction I sample. 2-A: reference standards of D-glutamate and D-phenylalanine at concentrations of 50 mM; 2-B: reference standards of D- and L-phenylalanine at concentration of 50 mM; 2-C: the reaction II sample

Reaction I: 4-methyl-2-oxovalerate + D-glutamate ↔ D-leucine + α-ketoglutarate

Reaction II: phenylpyruvate + D-glutamate ↔ D-phenylalanine + α-ketoglutarate

The yields of reactions were determined by monitoring the consumption of 4-methyl-2-oxovalerate or the accumulation of D-phenylalanine. Aliquot of deproteinized samples were analyzed by HPLC.

The chiral analysis of products of reactions I and II – D-Leu and D-Phe – was performed by HPLC using the reverse-phase C18 column with the UV detector set at 340 nm. Deproteinized samples were derivatized with Marfey's reagent (Sigma, USA) according to Pavkov-keller et al [1]. Briefly, 25 μl of Marfey's reagent (28 mM in acetonitrile) and 10 μl 1 M $NaHCO_3$ were

added to 10 µl sample and incubated at 50 °C for 2 h. The reaction mixture was cooled, and then the reaction was stopped by adding 3 µl of 4 M HCl and 10 µl of 100% ethanol.

HPLC analysis conditions.

Instrument:	ÄKTA Purifier (USA)
Column:	Zorbax Eclipse XDB-C18, 5 µm, 4.6 × 150 mm (Agilent, USA)
Column temperature:	25 °C
**The yields determination (Method A)**	
Eluent:	20 mM Na-phosphate buffer, pH 3.0, 15% MeOH
Flow rate:	1.0 ml/min
Injection volume:	20 µl
Detection:	UV, 210 nm
**The chiral analysis (Method B)**	
Eluent A:	0.1% trifluoroacetic acid in water
Eluent B:	0.1% trifluoroacetic acid in methanol
Flow rate:	1.0 ml/min
Gradient:	0-15 min: 20-70% Eluent B
Injection volume:	10 µl
Detection:	UV, 340 nm

Retention volumes of compounds

**Compound**	**V_{ret}, ml**	
**Method A**		
4-Methyl-2-oxovalerate	7.6	
D-Phenylalanine	4.3	
**Method B**		
	L-isomer	D-isomer
Leucine*	17.6	21.1
Phenylalanine*	17.0	19.9
Glutamate*	-	14.3

*After derivatization with Marfey’s reagent

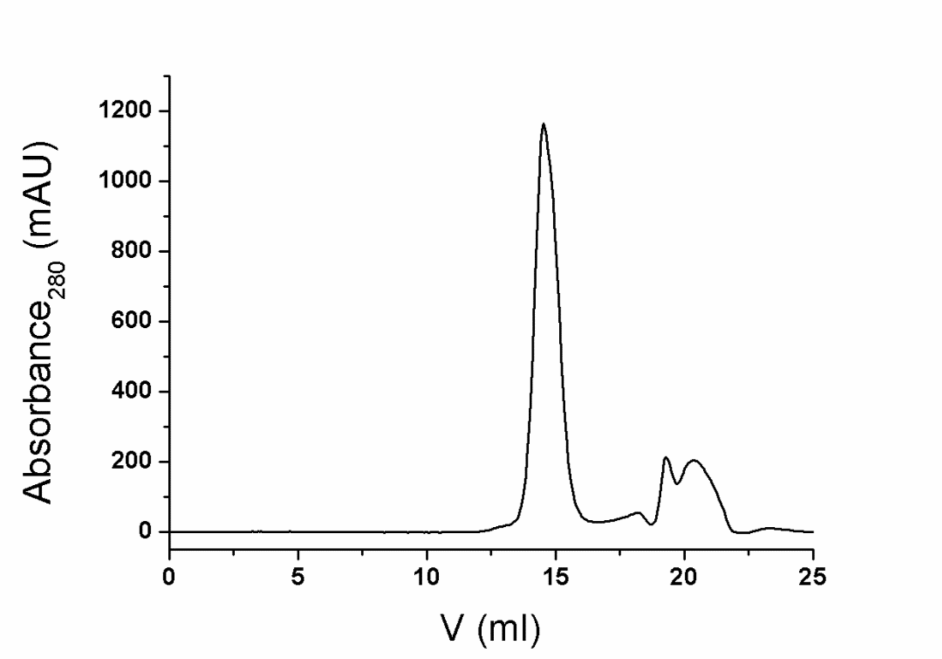

Figure S7. Gel filtration elution profile for AmicoTA. The major peak corresponds to the AmicoTA dimer, minor peaks correspond to low molecular weight compounds. Chromatography was carried out on a 24 mL Superdex 200 10/300 GL column equilibrated with 50 mM HEPES buffer, pH 8.0, containing 100 mM NaCl, 1 mM DTT, and 100 μM PLP.

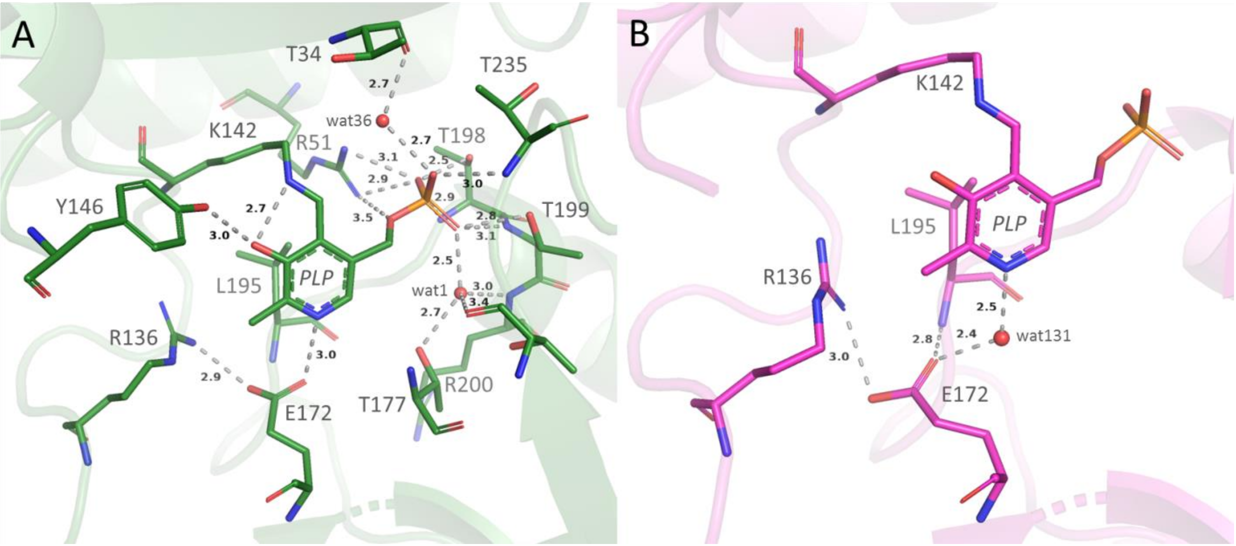

Figure S8. Binding of PLP molecule in the holoenzyme. Subunit A (A) and subunit B (B). Water molecules are shown as red balls. Distances are given in angstroms and shown as dashed lines.

.
